# Inducers of settlement and metamorphosis of the shrimp *Hippolyte inermis* Leach in *Posidonia oceanica*

**DOI:** 10.1038/s41598-019-48110-7

**Published:** 2019-08-26

**Authors:** Valerio Zupo, Mirko Mutalipassi, Francesca Glaviano, Anna Cecilia Buono, Antonio Cannavacciuolo, Patrick Fink

**Affiliations:** 1 Stazione Zoologica Anton Dohrn, Benthic Ecology Centre, Ischia, Punta San Pietro, 80077 Italy; 2 University of Cologne, Cologne Biocenter, Zülpicher Straße 47b, 50674 Köln, Germany; 3 Helmholtz Centre for Environmental Research, Department Aquatic Ecosystem Analysis, Brückstraße 3a, 39118 Magdeburg, Germany; 4 Helmholtz Centre for Environmental Research, Department River Ecology, Brückstraße 3a, 39118 Magdeburg, Germany

**Keywords:** Ecophysiology, Community ecology

## Abstract

Larvae of the caridean shrimp *Hippolyte inermis* persist in the plankton of the Mediterranean up to about one month. Since they need to reach appropriate coastal areas for their recruitment in seagrass meadows, we hypothesized that leaves of *Posidonia oceanica* or, alternatively, algae present in their epiphytic biofilms, might be physically recognised as target substrates and trigger larval metamorphosis and settlement. Chemical cues could improve the finding of suitable habitats for settlement. Thus, the effects of leaves of *P. oceanica* and biofilms of the diatom *Cocconeis scutellum parva*, seasonally abundant in the leaf epiphytic stratum, were investigated along with the effect of volatile organic compounds (VOCs) extracted from the epiphytic diatom. The physical induction with *P. oceanica* accelerated larval settlement, stimulating an earlier and faster metamorphosis of larvae. *C. scutellum parva* produced a weaker effect on settlement; however, diatom’s VOCs had evident influence and accelerated metamorphosis and settlement. We concluded that such chemical cues as the VOCs produced by epiphytic diatoms, reinforce the effect of physical cues for the identification of suitable settlement locations for this shrimp.

## Introduction

Moulting and settlement are key processes in aquatic environments, and the identification of suitable locations for recruitment strongly influences the fitness of invertebrates and the connectivity of their natural populations^1,2^. Settlement may be defined as either the attachment of a larval foot to the substrate (in case of sessile organisms) or the shift from a planktonic larval life to a benthic post-larva (in case of vagile organisms), preceded or followed by an irreversible developmental event of metamorphosis. Larval competency of natantia decapods, often preceding settlement, involves a noticeable resorption of plumose appendages and a significant elongation of eyestalks and walking legs, leading to the shape of a shrimp quite similar to adults of the same species^3^. The transition is associated with larval competency and metamorphosis of most decapods^4^. Since settling in an inadequate environment (higher probability of predation or scarce trophic resources) results in a failure of the reproductive event, invertebrate larvae evolved the ability to persist until suitable environmental conditions are reached^5,6^. The shrimp *Hippolyte inermis* Leach passes through a variable number of larval stages^7^. Each stage has a variable time of completion, according to the abundance and quality of food, temperature and other external stimuli. However, its larval development is completed consistently in about 40 days when fed on enriched *Artemia salina* nauplii and rotiferids, passing through 9 zoeal stages up to the last metamorphosis. The settlement percentage of cultured larvae, in our experimental conditions, varies from 65 to 95%^8^. Similarly, *Octopus* spp. paralarvae may last in a planktonic phase until appropriate environments are reached^9^, and various invertebrates developed the ability to recognize chemical^1^, biological^10–13^ or physical signals^14,15^ to identify appropriate substrates for settlement^4^ or the presence of adults of their own species^16^.

Habitat-specific environmental cues ensure successful larval recruitment for benthic invertebrates^17^. The presence of biofilms^18^ and in particular, specific metabolites released by bacteria and protozoans, are important settlement^19^ cues for larvae of sponges^20^ and an effect of chemical cues (e.g., humic acids, volatile infochemicals, ammonia and various ions, dissolved gases, organic compounds) on various invertebrate larvae^4,13,21^ was demonstrated. A nearly universal mechanism of settlement induction by bacterial cues has been proposed, able to strongly influence such different taxa of invertebrates as cnidarians^17,22^, bryozoans^23^, molluscs^16^, annelids^24^, echinoderms^19,25^, crustaceans^26^ and even ascidians^27^. A non-polar metabolite extracted from bacteria (Tetrabromopyrrole)^28^ is also responsible for partial or complete metamorphosis in coral planulae, which demonstrated a highly specific response to single infochemicals^29^. In addition, specific ions are recognised by larvae of the mussel *Mytilus galloprovincialis* to identify their target substrates^30^. Various coralline algae, as *Hydrolithon reinboldii* and *Neogoniolithon fosliei*, produce compounds that influence the settlement and further development of coral larvae^1^. The role of various algae-produced cues was also demonstrated for echinoderms^31^, corals^1^ and polychaetes^32^. Both physical and morphological cues influence the settlement of larvae, and the final choice depends on a multi-factorial strategy including surface topography^5^ and the availability of trophic resources, along with chemical signals^4^. Furthermore, chemical properties of substrates are important cues for habitat selection^33,34^. For these reasons, rather than investigating “if” a given species uses specific cues to improve the settlement process, we should strive to understand “which” cues influence the larval settlement, “how” they work and “why” they are employed, according to ecological and physiological needs of given invertebrates. However, a limited number of studies demonstrated the effects of physical and chemical cues on metamorphosis and settlement of marine crustacean decapods^35^, mainly on brachyuran crabs.

With this perspective we hypothesized that the shrimp *Hippolyte inermis*, strictly associated with the seagrass *Posidonia oceanica* in the Mediterranean sea^36^, might perceive and react to the presence of plant leaves. *H. inermis* is a stimulating subject of study, because of its close association with the leaves of the host seagrass and the trophic relationships with diatoms of the genus *Cocconeis*, influencing its ecology, physiology and sex reversal^37^. The leaf stratum of *Posidonia oceanica* represents its main habitat^38^ but shrimp larvae may be dispersed for a long time^7^ over wide areas, while their target *P. oceanica* meadows only cover narrow coastal areas^7,39^. However, competent larvae need to settle on seagrass leaves (normally distributed from 1 to 35 m depth)^40^ to survive, as they are delicate and strictly adapted to this environment^41^. Apart from plant leaf as physical cues, shrimp larvae could also perceive the presence of *Cocconeis* diatoms, typical epiphytes of *P. oceanica*. In fact, *Cocconeis* spp. are a key food item for *H. inermis*^42^ and the shrimp might improve its reproductive success if larvae could recognize its presence and select adequate locations for settlement, as it has been demonstrated for other decapod crustaceans. For example, substrate cues can shorten the instars and affect the subsequent nutritional condition in pueruli of the spiny lobster *Jasus edwardsii*^43^ and the Caribbean spiny lobster *Palinurus elephas*^2^. Accordingly, the main aims of this study were: (a) to test the effects of physical and chemical cues within the known time for post-larvae production^8^; (b) to test the effects of diatom films on the settlement of *H. inermis* and those of volatile organic compounds (VOCs) produced by *C. scutellum parva*^8,44^*;* and (c) to compare the proportion of larvae metamorphosing in presence of inducers and in controls, as suggested by Forward *et al*.^3^ for larvae of brachyuran crabs.

## Material and Methods

### Animal collection and maintenance

Ovigerous females of *Hippolyte inermis* were collected in fall 2017 in a *Posidonia oceanica* meadow off Lacco Ameno d’Ischia (Bay of Napoli, Italy. 40° 45′ 6″N; 13° 53′ 29″E) by trawling a plankton net over the leaves. Shrimp females were sorted on boat and transferred into plastic containers filled with clean water, until their transfer to the laboratory. Taxonomical identification was confirmed under the stereomicroscope. Shrimp females were individually reared in 2 L Erlenmeyer flasks containing 1.5 L of filtered and aerated seawater (at salinity 38.1 PSU), housed in a thermostatic chamber at 18 °C with a 12/12 day/night photoperiod. These conditions, routinely applied to our shrimp cultures, are close to those recorded in the field during the reproductive seasons. Seawater was filtered overnight by means of a canister filter (Eheim Classic 250 charged with synthetic foam and activated charcoal) and UV sterilized before use, to consistently provide a suitable rearing medium. Flasks were inspected daily for the presence of larvae and water was partially replaced with clean medium. Immediately after hatching, larvae were collected over a 60 µm mesh plastic filter, counted and individually transferred into 1 L flasks containing 800 mL of filtered seawater. *Artemia salina* nauplii (4 ind. mL^−1^) and *Brachionus plicatilis* (5 ind. mL^−1^) were added to the cultures as a food source. At the start of larval cultures, 80 larvae (pooled from several females) were stocked in each conical flask (1 ind/10 mL of seawater). Culture vessels were kept in the same thermostatic chamber at 18 °C, as specified above; the culture medium was renewed daily, and the number of larvae was recorded. Larvae were inspected daily under a stereomicroscope to follow the succession of zoeal stages^45^ up to the phases preceding the metamorphosis (zoeae VIII- IX), when plumose appendages were strongly reduced and the eyestalks started to be elongated. These characters, indicating the imminent reaching of a competent stage, occurred after 25–30 days^46^, according to temperature, season and feeding regimes. Larvae were transferred in glass plates after this stage, to start settlement experiments as further described.

### Experimental design and protocol

Settlement experiments, aimed at checking the reaching of final metamorphosis and the transformation of larvae into post-larvae (based on they morphology)^47^, were performed according to three treatments represented by (a) *Posidonia oceanica* leaves, (b) biofilms of *Cocconeis scutellum parva* and (c) diatom’s volatile organic compounds (VOCs). Each treatment was made up of 5 replicate 500 mL glass plates (14 cm diameter) filled with 400 mL of filtered seawater (pH 8.1, salinity 38.1 ppm, absence of measurable pollutants). Each plate, covered with a plastic lid and gently aerated, was stocked with a pool of 25 larvae. *Artemia salina* nauplii (5 ind mL^−1^) were provided during the test period and until the last zoea was settled as a post-larva. In addition, 5 mg of composed feed (Spirulina, Enriched *Artemia* and Micro-Granules by SHG, Ovada, Italy) were added daily to the bottom of each plate. Plates were kept in the same thermostatic chamber described above (12/12 photoperiod; 18 °C). Larvae assigned to each replicate treatment and its paired control were obtained from the same flask. Therefore, 50 out of the 80 larvae stocked in each replicate conical flask, were used for bioassays (25 for a replicate test; 25 for a paired control) while the remaining larvae were discarded. While paired controls were run according to the above experimental specifications, treatments were conducted in plates containing also a putative settlement inducer, either *P. oceanica* leaves, fresh *C. scutellum parva* biofilms or VOCs extracted from the diatoms.

### Leaf and diatom film treatments

Plates assigned to the treatment with seagrass leaves were provided with a 7 cm fragment of a *Posidonia oceanica* leaf. Only green parts were used, after rubbing leaves with cotton gauze to remove all epiphytes^48^. Leaves were replaced every 5 days to avoid leaching of decomposition products. The amount of leaf biomass used for this experiment simulates the presence of a *P. oceanica* meadow in the vicinity and it is aimed at testing the ability of larvae, to recognize its physical presence. Plates assigned to the treatment with diatom biofilms were pre-conditioned with an inoculum of *Cocconeis scutellum parva* (obtained from the strain collection of the Stazione Zoologica) 15 days prior to the beginning of the experiment. Diatoms were incubated in Guillard’s *f/2* medium^49^ to obtain a continuous biofilm with an average density of 35,000 cells. mm^−2^, as commonly measured on *P. oceanica* leaves^50^ in spring. Additional plates were inoculated with diatoms at 5-day intervals and used to transfer shrimps during the bioassay. Culture medium was replaced with sterilized natural seawater prior to the start of bioassays. The amount of infochemicals possibly emitted by intact diatom biofilms is quite low under these conditions, since this diatom produces wound-activated infochemicals only when mechanically damaged^44^. Thus, only a small fraction of diatoms naturally decaying (evaluated as 3–5% of the total biomass per day, according to our previous investigations) will produce infochemicals in the culture vessels. Diatom biofilms may hence be considered as physical cues, because *Cocconeis* spp. dominate the epiphytic layer of *P. oceanica* leaves in spring, during larval settlement^51^.

### VOC treatments

Plates assigned to the treatment “VOC” were prepared as described for controls but small amounts of volatile compounds were added daily to check if diatom chemical cues may influence the metamorphosis and settlement rates. In particular, we tested an ecological scenario represented by a leaf area of 2 cm^2^ covered with diatom biofilms and grazed by a herbivore, dispersed in the surrounding environment. As previously demonstrated^8^, the wounding by animal consumers of diatoms present in such a small area produces volatile compounds readily recognized by adults of *H. inermis*. We hypothesized that VOCs could be recognized by larvae as a settlement cue even at this low concentration, that decreases according to the distance from leaves. For this purpose, VOCs were extracted twice from sonicated diatom suspensions (2 × 40 mL) obtained from four 14-cm Petri Dishes covered with *C. scutellum parva* cultures. VOCs were concentrated by closed-loop stripping^52^ performed at 22 °C for 45 min and extracted on a Tenax TA cartridge, after addition of 10 g NaCl^53,54^. The cartridge was removed and eluted with 6 mL diethyl ether. The ether was gradually evaporated using nitrogen gas (N_2_, grade 5.0) and the residue re-dissolved into 300 µL of pure ethanol. Controls were prepared according to the same procedure, but stripping was performed on a sterile culture medium. All VOC samples and controls were stored at −80 °C until the start of bioassays. Every day, 1 µL of diatom VOC solution (corresponding to the VOCs produced by 2 cm^2^ of grazed diatom film) was added in each test plate, after a total change of the culture water, and 1 µL of VOC control solution was added in each control plate. This low dose was administered taking into account that a cue in the field should have maximum effect even in trace amounts^55^.

### Analysis of data

The abundance of larvae and post-larvae present in each plate was recorded daily for 15 days, to perform survival analyses by means of Kaplan-Meier estimates and detect influences of treatments on the temporal rates of settlement. Kaplan-Meier is a non-parametric method used to estimate the survival function from lifetime data. Mortality rates were recorded daily in each replicate and total mortality rates for each treatment were computed at the end of the experiment. One-way ANOVA was employed to test the significance of differences in mortality rates among treatments. Settlement curves were analysed as well by means of the Kaplan-Meier model^56^ and the significance of differences between treatments and their controls were tested by means of the Log-Rank (Mantel-Cox) technique using GraphPad Prism version 8.0.0, GraphPad Software (San Diego, California USA). In addition, since the abundance of newly metamorphosed juvenile shrimps may show daily fluctuations, the average number of post-larvae produced every 3 days in each plate was computed and compared in treatments and controls, to test the null hypothesis “No effect of the cue on the trends of production of postlarvae” using multiple t-tests, adapted for multiple comparisons using the Holm-Šídák method, with the assumption of homoscedasticity. Multiple t-tests were chosen in order to permit comparisons between couples of treatments vs. their paired controls, given the constraints of the experimental plan. The percentages of post-larvae produced at the end of the experimental time were computed as well and compared among treatments. Z-test on proportions was applied to check the significance of differences between the rates of post-larvae produced in induction treatments at each 3-day interval, and those produced in paired control plates. Finally, the average number of post-larvae produced after the half-time of the experiment, at the 9^th^ day of test, was calculated and compared between test plates and controls, using *Z*-test on proportions, to evaluate the effects of physical cues and volatile infochemicals on the process of settlement and the speed of maturation up to metamorphosis.

## Results

### Effects of physical and chemical cues

**Figure 1:**
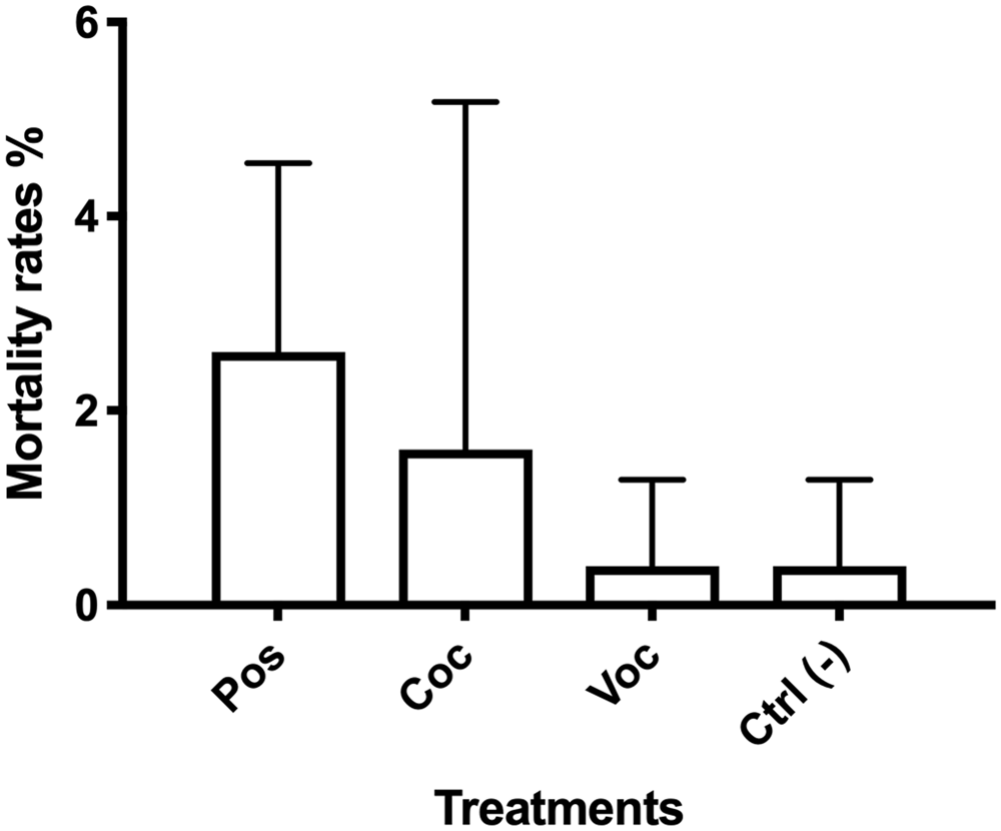
Average (on 5 replicates) of percent mortality rates over the total number of individuals, reached in 15 days of experiment in each treatment. Vertical bars indicate standard deviations among replicates.

**Figure 2:**
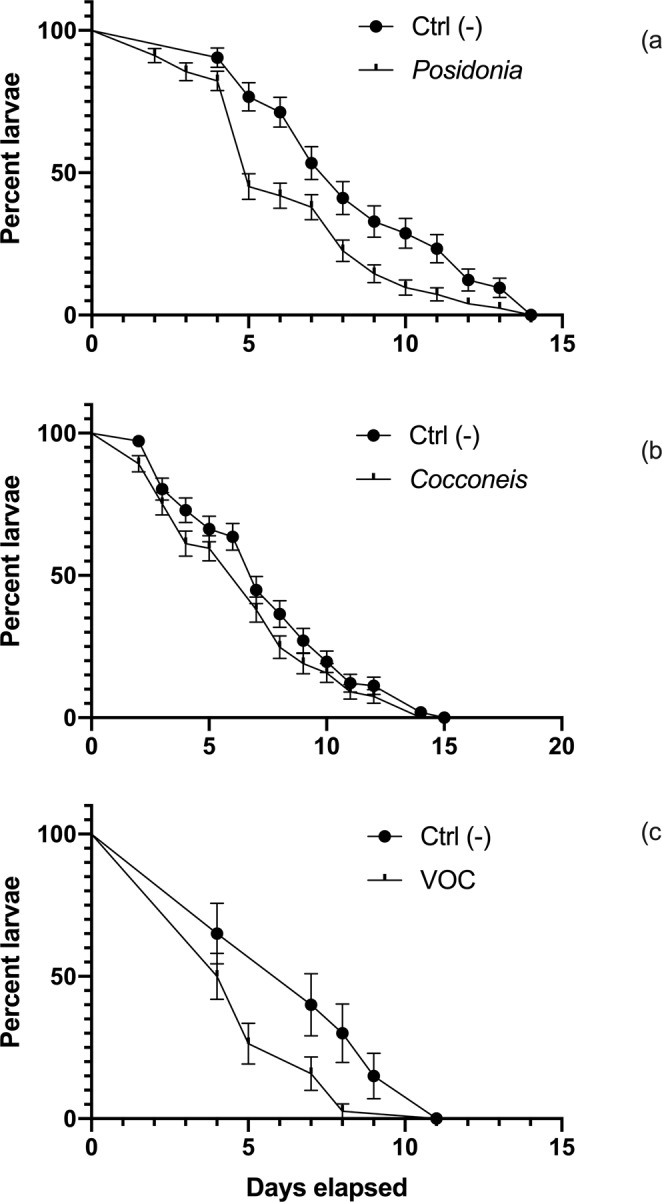
Percentages of larvae still in a planktonic phase in replicates of three treatments and their paired negative controls, during the first 15 days of test, plotted according to the Kaplan-Meier model. (**a**) Percentage of larvae in *Posidonia* treatment; (**b**) Percentage of larvae in *Cocconeis* treatment; (**c**) Percentage of larvae in VOC treatment. Vertical bars indicate standard deviations among 5 replicates.

In total, 36 ovigerous females were collected and reared up to the production of larvae. Larvae of cultured shrimp completed their growth within 25–30 days, reaching the stage of Zoea IX. At this stage, the induction experiments started. All treatments exhibited low mortality rates (Fig. 1), ranging between 0.4% ± 0.9 (in VOC treatments and controls) and 2.6% ± 1.9 (in the *Posidonia* treatment) of the total number of individuals. Differences in mortality rates among treatments were not significant (ANOVA, p > 0.05; Table 1) although *Posidonia* treatment exhibited slightly higher mortality rates than other treatments. Within 15 days, all treatments and controls exhibited similar trends of settlement and metamorphosis (Fig. 2a–c) and the reduction in the number of larvae in each treatment followed a logarithmically decreasing pattern. According to the Kaplan-Meier model, settlement rates in the *Posidonia* treatment and its controls differed significantly (at p < 0.001; Log-Rank test, Table 2). In contrast, settlement rates in *Cocconeis* treatment and its controls were not significantly different. Settlement rates differed significantly between the VOC treatment and its controls (at p < 0.01; Table 2). Less than 50% of larvae were still in a planktonic phase after 5 days, in most replicates, while most individuals had metamorphosed into juvenile shrimps. *Posidonia* treatments differed from their controls (Fig. 2a), but a decrease of metamorphosis was consistently observed after the 12^th^ day, as also recorded in the treatment *Cocconeis* (Fig. 2b). Variable rates of settlement and metamorphosis were observed according to treatments: 73.3% of larvae settled in *Posidonia* treatment, 95.1% in *Cocconeis* treatment and 98.6% in VOC treatment. In the VOC treatment the process was completed earlier than in other treatments (at the 10^th^ day) and a different pattern of settlement was exhibited by VOC-treated larvae in respect to their controls (Fig. 2c).

**Table 1:** Comparison of mortality by means of one-way ANOVA.

Data sets	A: *Posidonia oceanica*	B: *Cocconeis*	C: VOCs	D: CTRL	
Number of treatments: 4					
Number of values (total): 60					
	SS	DF	MS	r^2^	*p*
Treatment (between columns)	3.032	3	1.011	0.1038	0.1025
Residual (within columns)	26.17	56	0.4672		
Total	29.2	59			
Brown-Forsythe test					
F (DFn, DFd)	2,163 (3, 56)				
P value	0,1025				
P value summary	ns				
Are SDs significantly different (P < 0.05)?	No

**Table 2:** Settlement curves and time trends of post-larvae production in the treatments *Posidonia*, *Cocconeis* and VOC along with their controls analysed by means of Kaplan-Meier method.

*Posidonia* vs. Control		*Cocconeis* vs. Control		VOCs vs. Control	
Log-rank (Mantel-Cox) test		Log-rank (Mantel-Cox) test		Log-rank (Mantel-Cox) test	
Chi square	13,29	Chi square	2,56	Chi square	7,082
df	1	df	1	df	1
P value	0,0003	P value	0,1096	P value	0,0078
P value summary	***	P value summary	ns	P value summary	**
Are the survival curves sig different?	Yes	Are the survival curves sig different?	No	Are the survival curves sig different?	Yes
Gehan-Breslow-Wilcoxon test		Gehan-Breslow-Wilcoxon test		Gehan-Breslow-Wilcoxon test	
Chi square	15,27	Chi square	2,77	Chi square	4,866
df	1	df	1	df	1
P value	<0,0001	P value	0,0961	P value	0,0274
P value summary	****	P value summary	ns	P value summary	*
Are the survival curves sig different?	Yes	Are the survival curves sig different?	No	Are the survival curves sig different?	Yes
Hazard Ratio (logrank)	A/B	Hazard Ratio (logrank)	A/B	Hazard Ratio (logrank)	A/B
Ratio (and its reciprocal)	0,6412	Ratio (and its reciprocal)	0,8356	Ratio (and its reciprocal)	0,5927
95% CI of ratio	0,4848 to 0,8480	95% CI of ratio	0,6445 to 1,083	95% CI of ratio	0,3539 to 0,9926

### Temporal patterns of larval settlement

**Figure 3:**
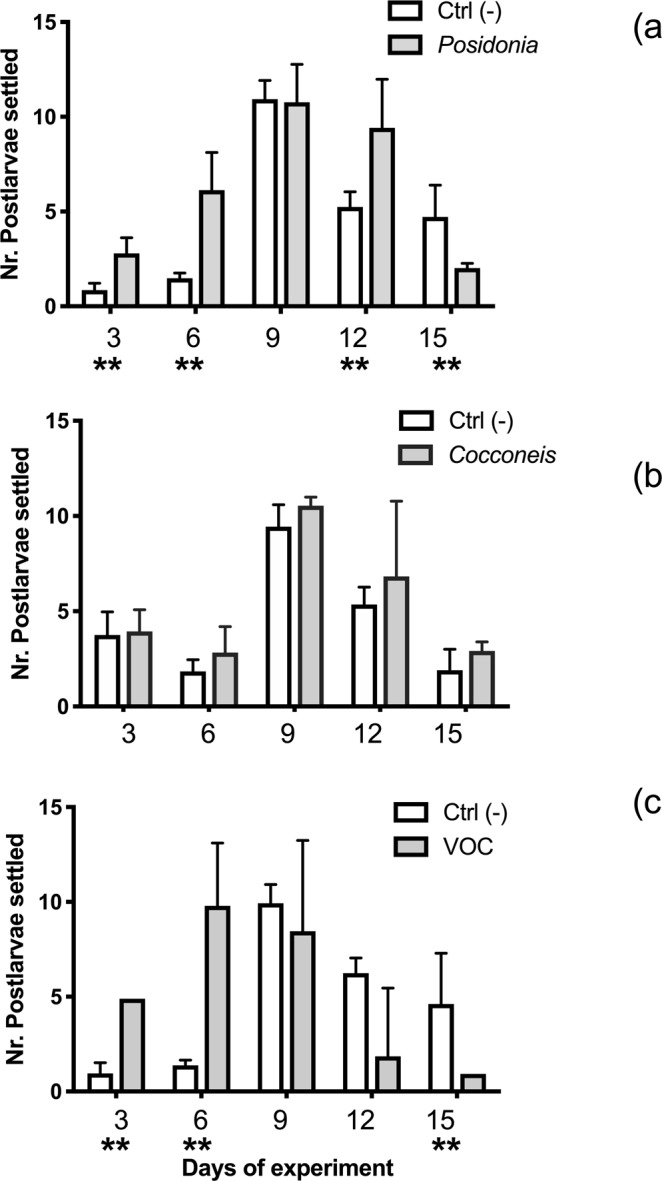
Average abundance of post-larvae settled every 3 days in each treatment and in paired controls and their standard deviations. (**a**) *Posidonia* and its control; (**b**) *Cocconeis* and its control; (**c**) VOC and its control. Asterisks indicate the significance of differences between tests and controls at 0.001 (**) or 0.05 (*).

The pools of post-larvae (PL) produced every three days (Fig. 3) in test plates were quite variable, ranging between 0.9 ± 0.35 and 11 ± 2.0 (Fig. 3a). Significant differences of PL production were detected among treatments (multiple t-tests; Table 3) but a gaussian pattern was consistently exhibited, with maximum values between the 6^th^ and 12^th^ day. In particular, the trends of PL production significantly differed between *Posidonia* and its control (Fig. 3a) and between VOC and its control (Fig. 3c), at most time-lags. In contrast, the differences between *Cocconeis* treatment and its controls were not significant (Fig. 3b) according to multiple t-tests (Table 3). *Posidonia* and VOC treatments showed different proportions of post-larvae produced in the first period of the experiment (*z*-test; p < 0.01), at the 3^rd^ and 6^th^ day, both producing a higher number of larvae metamorphosed as compared to the controls (Fig. 3a,c). After 6 days of treatment, the physical induction with *Posidonia* produced an increase of larval settlement and metamorphosis of 18.5% compared to the controls. The chemical induction with diatom VOCs triggered an increase of larval settlement and metamorphosis of 46.6% at the 6^th^ day, with respect to controls. The number of post-larvae produced in controls increased at the end of the experimental period (15 d) both in *Posidonia* and VOC treatments, indicating a delay in the completion of settlement as compared to induction treatments. The physical induction with *Cocconeis* biofilms did not produce any difference with respect to controls (Fig. 3b; Table 3).

**Table 3:** Results of multiple t tests performed on treatments and their controls performed on the average number of post-larvae produced every 3 days in each plate using the Holm-Šídák method, with the assumption of homoscedasticity.

Time	Significant	P value	Mean CTRL	Mean Pos	Difference	t ratio	df
**Posidonia**							
3	Yes	0,001	0,862	2,79	−1,929	4,772	8
6	Yes	0,001	1,476	6,127	−4,651	5,174	8
9	No	0,871	10,93	10,77	0,1668	0,1673	8
12	Yes	0,009	5,248	9,413	−4,164	3,458	8
15	Yes	0,007	4,724	2,019	2,705	3,582	8
**Cocconeis**							
3	No	0,810	3,752	3,938	−0,1854	0,2483	8
6	No	0,172	1,839	2,837	−0,9983	1,498	8
9	No	0,081	9,449	10,54	−1,094	1,999	8
12	No	0,440	5,362	6,833	−1,471	0,8124	8
15	No	0,097	1,908	2,917	−1,008	1,878	8
**VOC**							
3	Yes	0,000	0,9615	4,899	−3,937	15,86	8
6	Yes	0,000	1,376	9,797	−8,421	5,681	8
9	No	0,519	9,932	8,46	1,473	0,6747	8
12	No	0,029	6,248	1,852	4,396	2,66	8
15	Yes	0,015	4,624	0,9259	3,698	3,097	8

### Speed of metamorphosis in presence of inducers

**Figure 4:**
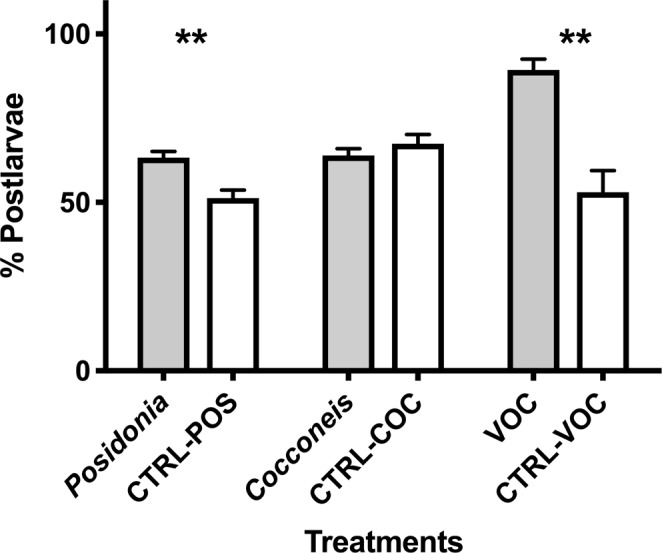
Average rates of post-larvae settled at the 9^th^ day of experiment in three treatments (*Posidonia*, *Cocconeis* and VOC) and in their controls (CTRL-POS, CTRL-COC, CTRL VOC, respectively) with their standard deviations. Asterisks indicate the significance of differences between tests and controls at 0.001 (**) or 0.05 (*).

The percentage of post-larvae settled and metamorphosed in the first half of the experiment (9 days) was 63.25% in the *Posidonia* treatment and 51.31% in its controls (Fig. 4). Similarly, 89.28% of larvae metamorphosed in the treatment VOC within the 9^th^ day of the experiment, as compared to 53.01% in its controls. In contrast, at the same time, 63.97% of larvae settled and metamorphosed in the *Cocconeis* treatment, compared to 67.41% in its controls (Fig. 4) which was not significantly different between treatment and control (p > 0.05). Overall, the production of post-larvae and settlement in induction tests and their controls significantly differed in VOC (p < 0.01) and *Posidonia* (p < 0.01) treatments but not in the test between *Cocconeis* and its controls.

## Discussion

A delayed metamorphosis of competent larvae, from a few days to several months, was demonstrated in various marine invertebrates in the absence of specific inducers^57^, associated with the recognition of adult habitats. The total time to complete the production of post-larvae was quite consistent among treatments, in accordance with previous observations^3^, indicating that in given conditions of culture the number of larval stages (from 5 to 9) and the persistence of each stage may be consistent, according to feed quality and temperature. The typical succession of zoeae was observed^7^, up to the production of competent larvae. Then metamorphosis and settlement continued according to a Gaussian trend during the next 15 days of the experiment. About 15% of post-larvae settled during the first and the last periods of test (the tails of a normal pattern), while most individuals metamorphosed between the 6^th^ and 12^th^ day of experiment, in all treatments. The exposure to *P. oceanica* leaves triggered a significant acceleration of this process. In fact, the treatments exposed to the presence of a green leaf yielded, on average, the settlement of 11.16% of post-larvae after 3 days of test, as compared to 3.84% of post-larvae settled in controls in the same period. An even higher difference was demonstrated at the time lag of 6 days (24.47 in *Posidonia* vs. 5.48 in controls). In the main period of competence (from the 6^th^ to 12^th^ day) the differences were less evident. This period also corresponds to the maximum rates of settlement, probably due to biological limitations for the species to delay metamorphosis before it becomes too costly^9,53^. Only in the last three days, at the completion of the process, the average number of post-larvae produced in controls was higher (18.48%) than the larvae produced in *Posidonia* treatments (8.04%). On the overall, the rates of individuals metamorphosed in the treatments containing *Posidonia* leaves were significantly higher than those exhibited by controls (80.43% *vs*. 64.41%). This trend is quite typical. It indicates that the presence of recognized cues triggers a shift to an earlier settlement and that, in the absence of inducers, larvae complete the development later, at the end of their period of competence^21^. Similar patterns were prompted by the addition of various ions (Li^+^, Cs^+^, RB^+^ and Ca^2+^) to the culture medium for the mussel *Mytilus galloprovincialis*. They produced a reduction (up to 25%) of the time for settlement, according to their concentrations^30^. This evidence stresses the importance of doses, since larvae may be sensible to various amounts of compounds having a direct or indirect effect in the settlement progression^55^. Thus, a different ratio of *P. oceanica* biomass *vs*. the volume of water could produce even stronger effects^58^. Similarly, various biofilms influence the settlement patterns and metamorphosis of *M. galloprovincialis*^12^, including diatoms, although the cue for molluscs was found to be mainly produced by the close association of algae and bacteria^59^.

The exposure of *H. inermis* to *Cocconeis scutellum parva* biofilms elicited less clear effects, as the rates of post-larvae production did not differ significantly in the presence of the diatom. However, volatile organic compounds released after wounding of *C. scutellum parva* cells had a marked effect on the metarmorphosis of *H. inermis*, which demonstrates that diatom VOCs may have an ecological function as infochemicals^29^. Nearly all known cases of chemical induction of larval settlement and metamorphosis involved substances adsorbed to the benthic substratum or sequestered in plants or bacteria encrusting substrates^60–62^. Decapod crustaceans have been often considered to dependent little on chemical cues for settlement and metamorphosis induction, probably because they retain their mobility after metamorphosis^57^ and waterborne compounds only rarely demonstrated to have an effect on these processes^55^. The choice to test this class of compounds was due to the demonstrated effect of diatom’s VOCs as infochemicals for adult shrimps^8^. Previous investigations demonstrated that plant VOCs were mainly recognized as warning signals, since they could indicate the presence of predators and they produce a negative chemotactic reaction in adults of *H. inermis*^44^. However, VOCs produced by the diatoms upon wounding triggered a different effect in shrimp larvae. This topic requires further attention, since infochemicals may change their “meaning” according to environmental contexts and life stages^44^. While in other invertebrates the larval receptors used to detect biofilms or bacterial cues are lost after metamorphosis^13^, in *H. inermis* the ability to recognize diatom VOCs is conserved in larvae and adults, but the “meaning” of the chemical signal as well as the elicited response changes according to life stages. *Cocconeis* diatoms send an attractive signal associated to gustatory receptors of adult shrimps, when consumed^51^, and a repulsive signal associated to olfactory receptors (indicating the presence of possible predators) when produced in low abundance in neighbouring areas^40^. In addition they produce a metamorphosis cue, in cooperation with the physical cues of *P. oceanica* leaves, recognized by shrimp’s competent larvae. The “thrifty” use of infochemicals and ancient receptors^4^, employed by the shrimp for a range of purposes, indicates an interesting area for future studies, aimed at detecting the evolution and co-evolution^63^ of chemical messages and their meaning throughout the life cycles of marine invertebrates.

This investigation confirms a substantial theoretical argument, suggesting the possibility for settlement and metamorphosis induction by waterborne VOCs produced by benthic diatoms. Diatom VOCs were previously known to play a role as infochemicals for adult *H. inermis*, but their signal has evidently also an importance for shrimp larvae searching for a feasible substrate. Both the seagrass leaves and VOCs extracted from an epiphyte diatom have been proven to be important for the shrimp^8^ and shortened its time of development to metamorphosis. This finding is remarkable, considering that multiple chemical cues may have a critical importance for settlement of invertebrates^59^. Thus, the association of *P. oceanica* physical influence and *C. scutellum* chemical cues might produce a stronger induction activity on larval metamorphosis, as observed for the mud crab *Panopeus herbstii*^9^. Also in this case, water-soluble compounds produced by microorganisms and other cues accelerated the metamorphosis of the crab megalopae. The tuning of metamorphosis according to environmental constraints (e.g., substrate recognition) may facilitate the settlement in adequate locations and explains the ecological success of this species in its seagrass environment. In addition, this study demonstrates that chemical messages, whose recognition was previously demonstrated only for adult individuals^8^, may be as important as physical cues to induce the metamorphosis of *H. inermis* and this finding could open interesting research opportunities for other species of decapods, for ecological and biotechnological purposes. In fact, larval biology issues and information on settlement and metamorphosis cues are essential for the development of aquaculture techniques as well as for the management of sustainable fisheries^35^.

Our results confirm the hypothesis that larvae of marine invertebrates do not settle randomly in any area, accepting the risk to survive only if they were lucky enough to encounter an acceptable site^13^. It demonstrates that also in vagile invertebrates, as marine shrimps, multiple cues improve the probability to select an ideal substrate to start their benthic life^21^. This finding reinforces the close relationship existing between shrimp and diatoms, co-evoluted in *P. oceanica* meadows, fundamental to facilitate its sex reversal in spring^51^. Thus, *H. inermis* may represent a good model to investigate the effect of metamorphosis cues during the search for a specific target environment, a seagrass having a narrow spatial distribution along the coastal areas of the Mediterranean.

### Compliance with ethical standards

This study was funded by the Flagship Project ModRes (SZN Flagship Project nr. 4) of the Stazione Zoologica di Napoli. Francesca Glaviano performed the bioassays funded by the flagship project ModRes of the Stazione Zoologica. Mirko Mutalipassi performed the bioassays while funded by a SZN Open University PhD project (V. Zupo as Director of Studies). All authors declare they have no conflicts of interest. This research involves the development of decapod crustaceans, not directly ruled by National Laws on animal welfare; however, all applicable international, national, and/or institutional guidelines for the care and use of animals were followed.

## Data Availability

All data sheets relative to the tests run are available at the Benthic Ecology Centre of Stazione Zoologica.
